# Towards a Combined Use of Geophysics and Remote Sensing Techniques for the Characterization of a Singular Building: “El Torreón” (the Tower) at Ulaca *Oppidum* (Solosancho, Ávila, Spain)

**DOI:** 10.3390/s21092934

**Published:** 2021-04-22

**Authors:** Miguel Ángel Maté-González, Cristina Sáez Blázquez, Pedro Carrasco García, Jesús Rodríguez-Hernández, Jesús Fernández Hernández, Javier Vallés Iriso, Yolanda Torres, Libertad Troitiño Torralba, Lloyd A. Courtenay, Diego González-Aguilera, Serafín López-Cuervo, Julián Aguirre de Mata, Jesús Velasco Gómez, Marco Piras, Andrea di Filippo, José Yravedra, Maximiliano Fernández Fernández, Teresa Chapa, Gonzalo Ruiz Zapatero, Jesús R. Álvarez-Sanchís

**Affiliations:** 1 Department of Topographic and Cartography Engineering, Higher Technical School of Engineers in Topography, Geodesy and Cartography, Universidad Politécnica de Madrid, Mercator 2, 28031 Madrid, Spain; y.torres@upm.es (Y.T.); s.lopezc@upm.es (S.L.-C.); julian.aguirre@upm.es (J.A.d.M.); jesus.velasco@upm.es (J.V.G.); 2 Department of Cartographic and Land Engineering, Higher Polytechnic School of Ávila, University of Salamanca, Hornos Caleros 50, 05003 Ávila, Spain; u107596@usal.es (C.S.B.); retep81@usal.es (P.C.G.); j.f.h@usal.es (J.F.H.); la_courtenay@usal.es (L.A.C.); daguilera@usal.es (D.G.-A.); 3 Gran Duque de Alba Institution, Diputación Provincial de Ávila, Paseo Dos de Mayo, 8, 05001 Ávila, Spain; ltroitin@ucm.es (L.T.T.); maxifernandezav@hotmail.com (M.F.F.); jralvare@ghis.ucm.es (J.R.Á.-S.); 4 Department of Environment, Land and Infrastructure Engineering, Politecnico di Torino, 10129 Torino, Italy; marco.piras@polito.it; 5 Department of Natural Resources & Environmental Engineering, University of Vigo, Maxwell s/n, 36310 Vigo, Spain; 6 Department of Prehistory, Ancient History and Archaeology, Complutense University of Madrid, Prof. Aranguren s/n, 28040 Madrid, Spain; jesusrodriguez@ucm.es (J.R.-H.); j.valles@ucm.es (J.V.I.); joyravedra@hotmail.com (J.Y.); tchapa@ghis.ucm.es (T.C.); gonzalor@ghis.ucm.es (G.R.Z.); 7 Centro de Asistencia a la Investigación (C.A.I.) de Arqueometría y Análisis Arqueológico, Complutense University of Madrid, Prof. Aranguren s/n, 28040 Madrid, Spain; 8 Department of Human Geography, Complutense University of Madrid, Prof. Aranguren s/n, 28040 Madrid, Spain; 9 Department of Civil Engineering, University of Salerno, Via Giovanni Paolo II 132, 84084 Fisciano, Italy; anddifilippo1@unisa.it; 10 Department Sciences of Communication and Sociology, Faculty of Communication Sciences, Rey Juan Carlos University, Camino del Molino s/n, Fuenlabrada, 28943 Madrid, Spain

**Keywords:** *oppidum*, vettones, remote sensing techniques, geophysical approaches

## Abstract

This research focuses on the study of the ruins of a large building known as “El Torreón” (the Tower), belonging to the Ulaca *oppidum* (Solosancho, Province of Ávila, Spain). Different remote sensing and geophysical approaches have been used to fulfil this objective, providing a better understanding of the building’s functionality in this town, which belongs to the Late Iron Age (ca. 300–50 BCE). In this sense, the outer limits of the ruins have been identified using photogrammetry and convergent drone flights. An additional drone flight was conducted in the surrounding area to find additional data that could be used for more global interpretations. Magnetometry was used to analyze the underground bedrock structure and ground penetrating radar (GPR) was employed to evaluate the internal layout of the ruins. The combination of these digital methodologies (surface and underground) has provided a new perspective for the improved interpretation of “El Torreón” and its characteristics. Research of this type presents additional guidelines for better understanding of the role of this structure with regards to other buildings in the Ulaca *oppidum*. The results of these studies will additionally allow archaeologists to better plan future interventions while presenting new data that can be used for the interpretation of this archaeological complex on a larger scale.

## 1. Introduction

Common practice in archaeology requires extensive documentation of a given region of interest (ROI), prior to any kind of intervention. In this sense, geotechnologies provide essential benefits for archaeology as they employ an array of non-destructive techniques for the characterization of heritage assets. From this perspective, remote sensing and geophysical methodologies allow for the geometric characterization of the surface and buried elements of different archaeological sites. This provides significant advantages when: (i) analyzing and identifying the most interesting areas to excavate; (ii) planning an excavation; (iii) optimizing material and human resources, which are often limited; (iv) increasing capacities in decision-making tasks, considering possible day-to-day problems while additionally providing a broader understanding of the ROI; (v) carrying out high-resolution documentation of the site, providing a means of facilitating both site conservation and interpretation. It is important to clarify that, although geophysical methodologies can be conceived as remote sensing techniques, it is sometimes convenient to separate these two terms. While applied geophysics mainly focuses on the subsurface, typical remote sensing techniques have the ability to accurately image the Earth’s surface with high-resolution investigations carried out by means of terrestrial, airborne, or satellite-based platforms.

The current use of geophysical prospecting methods includes numerous techniques dedicated to the study of the subsoil. These methods are widely used in archaeology for the characterization of soil composition and stratigraphy and for defining the geometry of underlying structures or the surrounding geology of the area under study [1,2,3,4,5,6,7,8]. As a general rule, the presence of different types of anthropic activities results in a significant modification of the properties and integrity of the ground. These geophysical features allow for the evaluation of differences between the physical properties of archaeological remains and the soils that surround them. Different geophysics techniques (magnetometry [8,9,10,11], ground penetrating radars (GPR) [8,12,13,14], or electrical resistivity tomography (ERT) [8,14,15,16], among others) provide images of the different features via 2D maps, 3D maps, or in a sequence of 2D profiles. In this sense, GPR is specially designed to capture the presence of permittivity contrasts and ERT is able to differentiate electrical conductive layers, while magnetometry is used to quantify the strength or direction of the magnetic signal from the ground and those elements found within [9,10,11,12,13,14,15,16]. Furthermore, it must be pointed out that results in deep horizons are possible from any technique (even GPR), but are not useful for delineating archaeological features. Likewise, for the localization of specific elements, GPR and magnetometry present the most precise results. ERT, however, presents lower resolution (if the electrode separation and survey spacing is not sufficiently small) and has a longer data acquisition time [9,10,11,12,13,14,15,16].

From the use of the above techniques (prior to the excavation process and also during it), excavation teams can focus their interest and resources on the areas where the best results are expected to be found [1,17,18], optimizing both resources and energy. On the other hand, the use of remote sensing for the documentation and analysis of archaeological sites has represented a great benefit within the field of cultural heritage. These techniques have considerably improved the precision of these processes, additionally allowing for the integration of new research methodologies and more exhaustive archaeological analyses [19,20,21,22]. An additional positive aspect is that these techniques enable a more graphic and visual dissemination of results, adapted in accordance with the researcher’s needs [23,24,25,26].

The primary remote sensing methods applied in archaeology currently consist of range-based methods (static/dynamic laser scanner) [23,26,27,28] and image-based methods (photogrammetry/image-based modelling) [24,29,30]. Likewise, techniques such as aerial photogrammetry or airborne light detection and ranging (LiDAR) and terrestrial photogrammetry or laser scanners are essential for the documentation of open spaces [31,32,33,34,35,36]. These techniques also allow obtaining different products derived from 3D point clouds, such as: 3D models, orthophotos, digital elevation models (DEM), digital surface models (DSM), digital terrain models (DTM), or other useful types of analyses. More information about each geomatics’ procedures can be found in previous authors’ research [14].

The present study describes a multidisciplinary methodological approach to carrying out a surface and underground geometric characterization of the ruins of a large building known as “El Torreón” (the Tower) located in the archaeological site of Ulaca (ca. 300–50 BCE). There is a current lack of investigation that deals in depth with the location, characteristics, and possible function of “El Torreón”. The research at Ulaca has a long but discontinuous history [37,38]. Since Ballesteros [39] provided the first news about the site, followed by the first exhaustive description by Gómez-Moreno [40], different researchers both national and international have tried to develop research into this site. Under this premise, researches have addressed the exploration of its massive defensive system, its urban structure, the monumental constructions included within the limits of the *oppidum*, the associated necropolis, as well as the site’s context and role among the pre-Roman settlements of the Amblés Valley [41,42,43,44,45,46,47,48,49]. Through the combination of remote sensing with geophysical techniques, the present analysis provides a new perspective for the understanding of particular structures within the late Iron Age *oppidum* of Ulaca. The Tower’s function will then be discussed throughout this study and the use of a certain geophysical method will be key to searching for cuts into the surrounding bedrock or natural springs. The importance of these formations derives from the fact that they could be an indication that the Tower was located in that place for strategic water use. Data obtained serves as a useful guide for the planning of future archaeological interventions aimed at improving the understanding of the global environment of this archaeological complex. Within this context, the paper has been organized as follows: Section 2 introduces the case study and defines the materials and methods used for characterization processes; Section 3 describes the experimental results obtained; Section 4 exposes the discussion and finally some conclusions obtained from the present work are drawn within Section 5.

## 2. Materials and Methods

### 2.1. The Ulaca Oppidum (Ávila, Spain)

**Figure 1:**
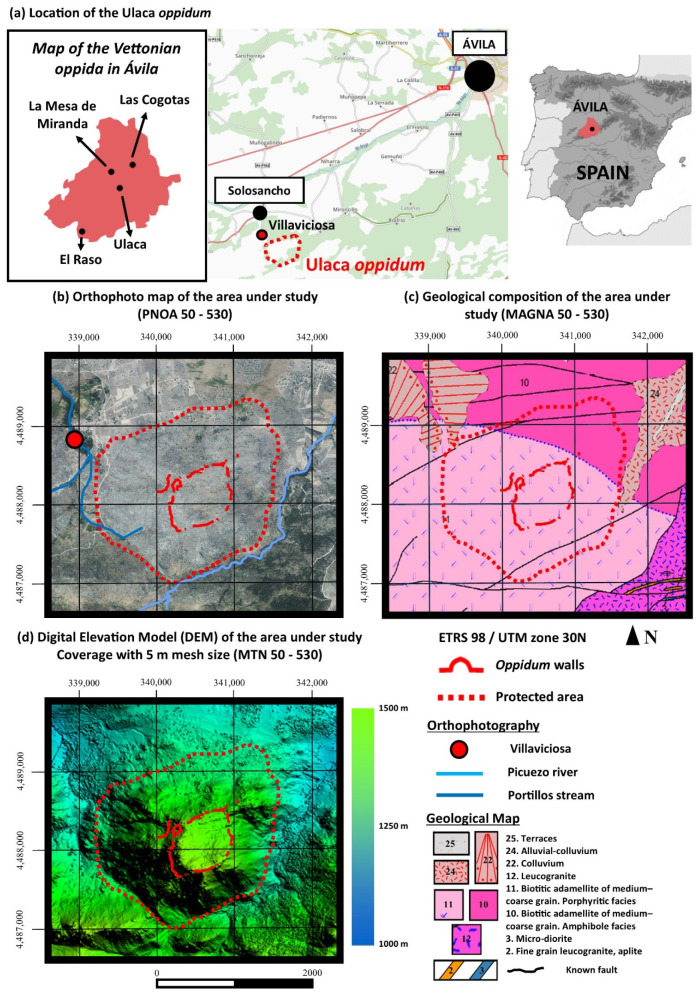
Location and geology of the Ulaca *oppidum* (Villaviciosa, Solosancho, Ávila, Spain).

The Vettones were one of the most remarkable populi in Celtic Iberia [50,51,52,53]. Texts addressing these populations from classical Greek and Roman literature described them occupying the territory of the current provinces of Ávila and Salamanca, as well as parts of Zamora, Cáceres, and Toledo. Around 400 BCE the earliest urban settlements or *oppida* (in Latin terminology) arose among these communities throughout the west of the peninsular Meseta (plateau). These *oppida* generally occupy strategic places with natural defenses that were additionally reinforced by powerful walls, upright stone bands (chevaux-de-frise), and ditches [54]. This type of urban nucleus could house several hundred or thousands of people, dedicated mainly to economic activities associated with agriculture and livestock. The importance of the latter is evident as seen through the presence of more than 400 sculptures of bulls and pigs across the west sector of the Iberian Central Meseta. These stone figures, popularly known as “verracos” (boars), are an important icon in the modern-day cultural heritage of these areas [55]. The most famous *oppida* in the province of Ávila are: Ulaca, Las Cogotas, La Mesa de Miranda, and El Raso (Figure 1a).

The Ulaca *oppidum* is located close to the village of Villaviciosa (Solosancho, Province of Ávila, Spain) (Figure 1a). This site was occupied towards the end of the Iron Age (ca. 300–50 BCE) by a community of Vettones of around 1500 inhabitants, becoming one of the largest fortified settlements in the Iberian Peninsula [56]. The creation of this urban nucleus converted this site into the most significant town of the Amblés Valley, where two other large fortified settlements are known; “Las Cogotas” in Cardeñosa and “La Mesa de Miranda” in Chamartín [57] (Figure 1a). Both sites are around 25 km away from Ulaca, but there is no intervisibility between them due to the rugged nature of the area. The Ulaca *oppidum* is located on an extensive granite plateau-like summit at about 1500 m above sea level, on top of a hill raised about 250 m above the surrounding terrain [41] (Figure 1b–d). The landscape of Ulaca stands out by the presence of numerous granite rocks with associations of various morphologies (such as balancing rocks or boulders), derived from the alteration or weathering of the initial granite bodies (Figure 1c). The hill where the *oppidum* is located is embedded between the Picuezo River and Los Portillos Stream and is protected to the south by the peaks of the Sierra de la Paramera, while to the north it opens to the wide Amblés Valley (Figure 1a). In 1995, Ulaca was declared “Bien de Interés Cultural” (Asset of Cultural Interest); the highest level of protection for historical heritage in Spain [58,59]. The protected archaeological zone includes the *oppidum* and its cemetery. This protected area refers to the part of the site which is under special protection measures by the local administrations.

**Figure 2:**
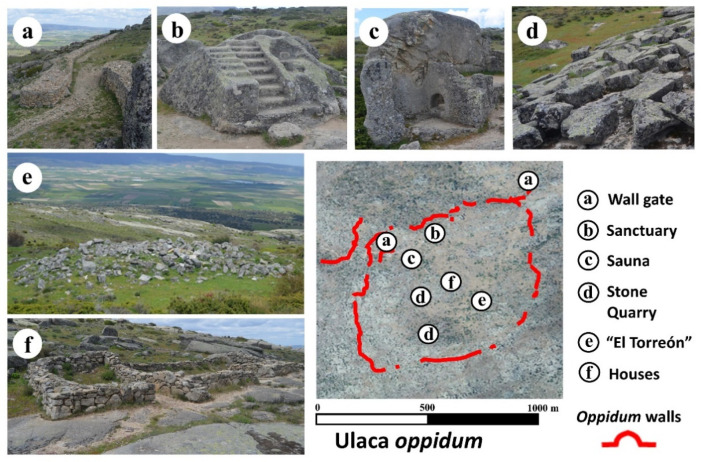
Principal monuments found within the Ulaca *oppidum.*

Ulaca stands out from the rest of the Vettone settlements for its size, covering more than 70 ha, defended by more than 3000 m of walls (Figure 2a) [42] and its well-preserved structures, some of them exceptional in the Celtic world, such as a rock sanctuary (Figure 2b) [41], a sauna semi-excavated in the rock (Figure 2c) [43], and some granite quarries (Figure 2d) [60]. Additionally, archaeological excavations from the last two decades have revealed the location of an area of artisan workshops and a cemetery, located on the northern slope of the *oppidum* and outside of the walled enclosure [44,45]. Despite the extensive knowledge of the site, there are still some crucial aspects to be determined. One of these enigmatic elements is the presence in the southern sector of a voluminous building in ruins known as “El Torreón” (Figure 2e). This construction, which according to Gómez-Moreno measures 15 m × 10.3 m [40], is built with large granite blocks, clearly different from the other > 250 domestic structures that have been identified scattered around diverse areas of the settlement (Figure 2f). “El Torreón” has no parallels in the entire Vettonian area and has traditionally been interpreted as a defensive watchtower [38] (p. 415) or as a public building [47].

### 2.2. Integrative Characterization of “El Torreón” by Means of Remote Sensing and Geophysical Approaches

#### 2.2.1. Aerial Photogrammetry

**Figure 3:**
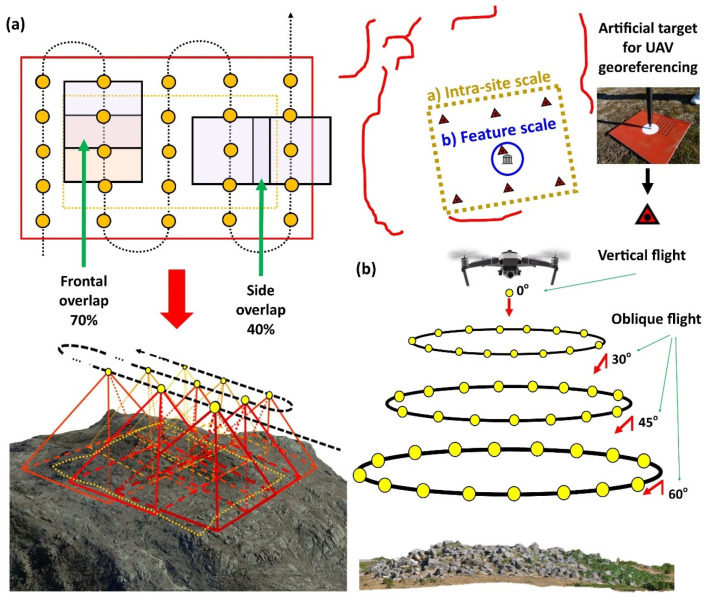
(**a**). Flight plan following a parallel photographic shooting protocol for the elaboration of the cartography for the ROI. (**b**). Flight plan following an oblique/convergent photographic shooting protocol for the documentation of “El Torreón” ruins.

Under the premise of defining the cartography and documenting the ROI surrounding the ruins of “El Torreón”, different photogrammetric techniques have been used. These techniques allow for the construction of 2D and 3D metric products from aerial images collected from a drone. In the present study, a DJI Mavic 2 Pro (Figure 3) was used to capture the images from two flights applying different protocols.

Firstly, for the elaboration of the area’s cartography, photographs taken from a drone were performed following a parallel flight plan. Images were captured with image overlaps of 70% (frontal overlap, with respect to the flight direction) and 40% (side overlap) (Figure 3a). The flight was then planned, taking into account drone model, location, and extent of the land, orography, and meteorological conditions (no rain or predominant winds), since Ulaca is located at high altitudes where strong winds are predominant. Specific software called Flight Planner was used for planning the flight [61]. In addition to this, coordinates were surveyed with a global navigation satellite system (GNSS) instrument (Topcon GR-5), with an accuracy of ±1 cm and targets uniformly distributed throughout the ROI. These targets were easily identifiable in each of the photographs, enabling us to solve the external orientation and georeferencing of the consequent analyses (Figure 3a). Finally, 300 images taken at a flight height of 80–85 m were obtained, occupying an area of 3 ha, estimating a ground sampling distance (GSD) value of 4.5 cm.

Secondly, for the documentation of “El Torreón”, photographs were taken from a drone following an oblique/convergent photographic shooting protocol. Photos were taken ensuring a proper overlap among images (around 80–90%), with separation among them of 10 to 15° and maintaining a constant distance from the building. In order to perform this task, a series of photographs were taken in a single flight following a circular sequence (360°) centering the point of view of each image towards the center of the building, flying in a circular pattern at three heights above the ground (15 m with a 60° camera inclination, 30 m with a 45° camera inclination, and 60 m with a 30° camera inclination) (Figure 3b). Finally, a total of 94 images were obtained, occupying an area of 0.6 ha, estimating an average GSD value of 1.5 cm.

Once drone flights were executed, images were processed with photogrammetric reconstruction software. This reconstruction was made using the open source software GRAPHOS [62,63].

#### 2.2.2. Geophysical Methods

##### Magnetometry

Magnetic methods are probably the most used geophysical techniques for numerous practical approaches. This method is capable of detecting deviations from the normal geomagnetic field due to the presence of certain minerals or remains magnetization. In the archaeological field, the information derived from magnetic prospecting is especially valuable for the identification of different structures, such as ditches, pits, or walls [9,10,11].

In the surveys presented here, a global enterprise management system (GEM system) consisting of the following units was used:

- Mobile unit (walking displacement mode), GEM Gsm-19 proton Overhauser magnetometer (0.022 nT of sensitivity) with internal global navigation satellite system (GNSS) of simultaneous register of the magnetic field. Measurement range of 20,000 to 120,000 nT and sampling interval of 0.2 s.
- Base (whose coordinates are known), potassium magnetometer, GEM Gsmp-40, with a sensitivity of 0.002 nT and a measurement range of 20,000 to 100,000 nT, with a sampling interval of 1 s. This is employed for any daytime corrections in the mobile unit when required.

For the present study, the magnetic prospection was carried out in the area surrounding “El Torreón” with more than 48 registers or routes around the study area, spread over numerous research profiles, performing more than 3.89 km of measurements. The covered area has an approximate surface area of around 4000 m^2^, with a spatial resolution of 0.2 m in “y” and 1 m in “x” and absolute accuracy of 0.1 nT. The mentioned spatial resolution was obtained from the post-processing of GNSS data.

Once the magnetic prospecting in the field was performed, some steps were required before obtaining the results. Data processing is based on the use of mathematical algorithms to provide significant information to be subsequently interpreted such as diurnal correction, polo reduction, and VOXI Earth Modelling algorithm. For this purpose, filters are commonly used to attenuate the noise of the signal or to enhance the most interesting parts of it. Two main processing phases were applied:

- Preprocessing: the aim is to correct the errors in the signal associated with data acquisition (sudden movements and external sources). After this phase, the daytime correction is also performed.
- Processing: geological characterization of the ground based on the signal treatment through Voxi Earth Modelling algorithm of Oasis Montaj Software.

Preprocessing phase includes several corrections (diurnal, interpolation of magnetic data or pole reduction, among others) required to obtain the ground final results [9,10,11].

##### Ground Penetration Radar (GPR)

GPR geophysical technique is based on the emission of short-duration electromagnetic impulses. These are reflected and detected by the receiving antenna when an object or a discontinuity area is intercepted [64]. In this sense, only a part of all the energy that arrives at the object is reflected, depending on the electrical properties of the object. The remaining energy continues until additionally reflected by a new object. This repeats until total absorption occurs.

Data must be post-processed, both for their georeferencing and for the cleaning of noise that prevents the observation of reflections of interest. Thanks to this technique, it is possible to obtain flat and three-dimensional representations from data organized according to vertical sections or radargrams [65,66].

In this research, the GPR system used was a commercial device of the Ingegneria Dei Sistemi (IDS) Stream X model, to cover a prospecting area of around 2800 m^2^. This equipment consists of a multichannel antenna with a nominal frequency of 600 MHz. The antenna consists of 12 dipoles (and 11 channels) with a polarization in vertical transmit and vertical receive (VV) and a spacing of 8 cm. This allows performing 11 parallel sections with very little spacing at a higher acquisition speed, enabling the coverage of large areas in much less time and with high resolution while remaining accurate in its 3D representations and measurements. Field data acquisition consisted of making rectilinear transects. The starting and ending point of each transect was georeferenced by using a GNSS instrument with accuracies of ±1 cm (Topcon GR-5 model). This was performed so that anomalies or interpretations obtained from the study could be located with greater accuracy. The acquisition characteristics for each radargram consisted of 87 scans/s of 12 samples/s with a 64 ns window and a horizontal resolution of 12 scans/m. This was performed using the IDS K2 software.

During data collection, some limitations were detected when acquiring the data with this technique. This was due to the existence of areas of high humidity or obstacles encountered that prevented the antenna from advancing. These obstacles included accumulations of rocks, debris, shrubs, trees, among other impediments that prevented the antenna from advancing while remaining attached to the ground. Moreover, GPR was not able to obtain information about the interior area of the ruined building.

Field data were processed using a series of algorithms or digital filters in which the signal was amplified and “cleaned”. The objective of this was to obtain results that can be interpretable by a specialist or technician. The results obtained depended on several factors that condition the transmission of energy, such as the moisture content of the soil, the amount of clay, or the homogeneity of the medium. In unfavorable conditions with high content of clays and humidity, energy is attenuated and the estimated depths are not reached. In heterogeneous media, the signal is scattered and poor quality results can be obtained.

The raw data obtained in the field with GPR, together with the GNSS data, were processed using the GPR-Slice software (GPR Slice web [67]). Processing sequences vary slightly according to the area but are almost constant. The velocities of the medium were calculated using methods involving the size of the hyperbolas according to position [13], as well as the average values, considering the soil to be homogeneous with similar characteristics and small variations in grain size. The results obtained or radargrams represent wave amplitude versus time of travel for the electromagnetic pulse. Through estimating the electromagnetic velocity of the ground (0.103 m/ns), the depth of localized reflections can be inferred.

In total, 75 transects were made in various phases and areas. These were adjusted according to the obstacles encountered in the local environment. A total of 825 2D radargrams were obtained. Once processed, these radargrams allowed for the generation of different 3D block anomaly maps. The mentioned processing was made using and fitting 0 ns, wobble correction, gain adjustment, background removal, bandpass filter, and Hilbert transformation. Three-dimensional blocks were obtained by interpolation with the inverse of distance method (cell size 8 cm in x and y axis and 7 cm in the z axis, without overlap).

## 3. Results

### 3.1. Definition of the External Geometry

**Figure 4:**
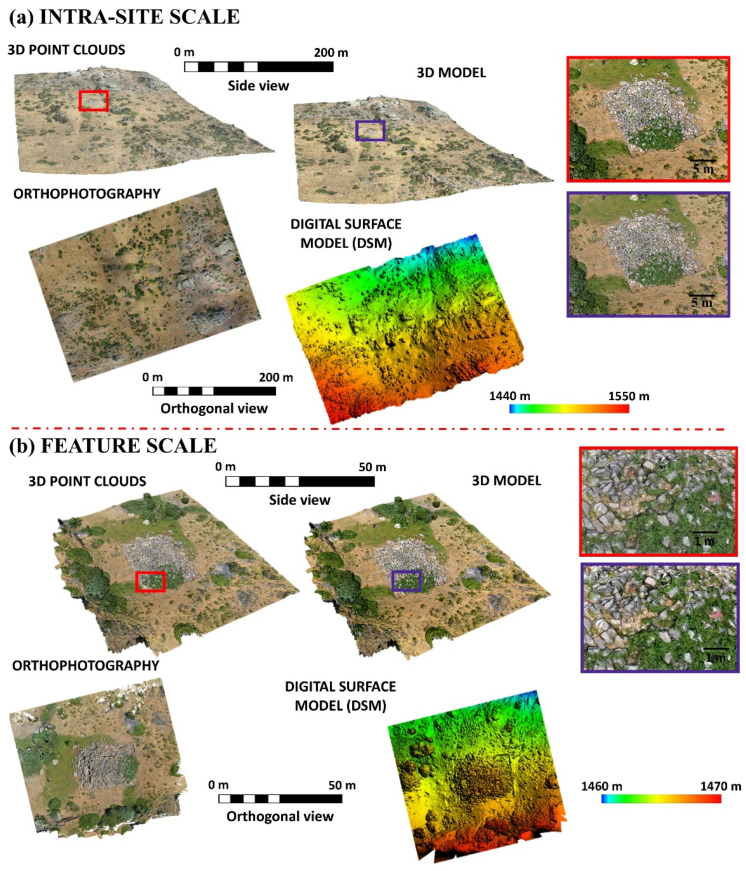
(**a**) Results of the parallel photographic shooting protocol (Figure 3a). (**b**) Results of the oblique/convergent photographic shooting protocol (Figure 3b).

Thanks to GRAPHOS, the dense point cloud and 3D model (mesh), DEM and orthophoto were generated for the surrounding areas of “El Torreón” (final results: GSD of 4.4 cm, root mean square error (RMSE): 3.2 cm, resulting from the residual errors at control points (considering the use of 10 control points obtained with GNSS)). From this, the DSM (GSD: 8.8 cm) and orthophoto (GSD: 2.3 cm) were generated (Figure 4a). Likewise, photogrammetry was also able to successfully capture the structure of “El Torreón” itself (point cloud and 3D model (mesh)) (final results: GSD of 1.5 cm, RMSE: 1.2 cm, resulting from the residual errors at control points (considering the use of eight control points obtained with GNSS)). In the same way, the DSM (GSD: 1.4 cm) and orthophoto (GSD: 0.6 cm) were generated (Figure 4b).

#### 3.1.1. Characterization at an Intra-Site Scale (around the Ruined Building)

**Figure 5:**
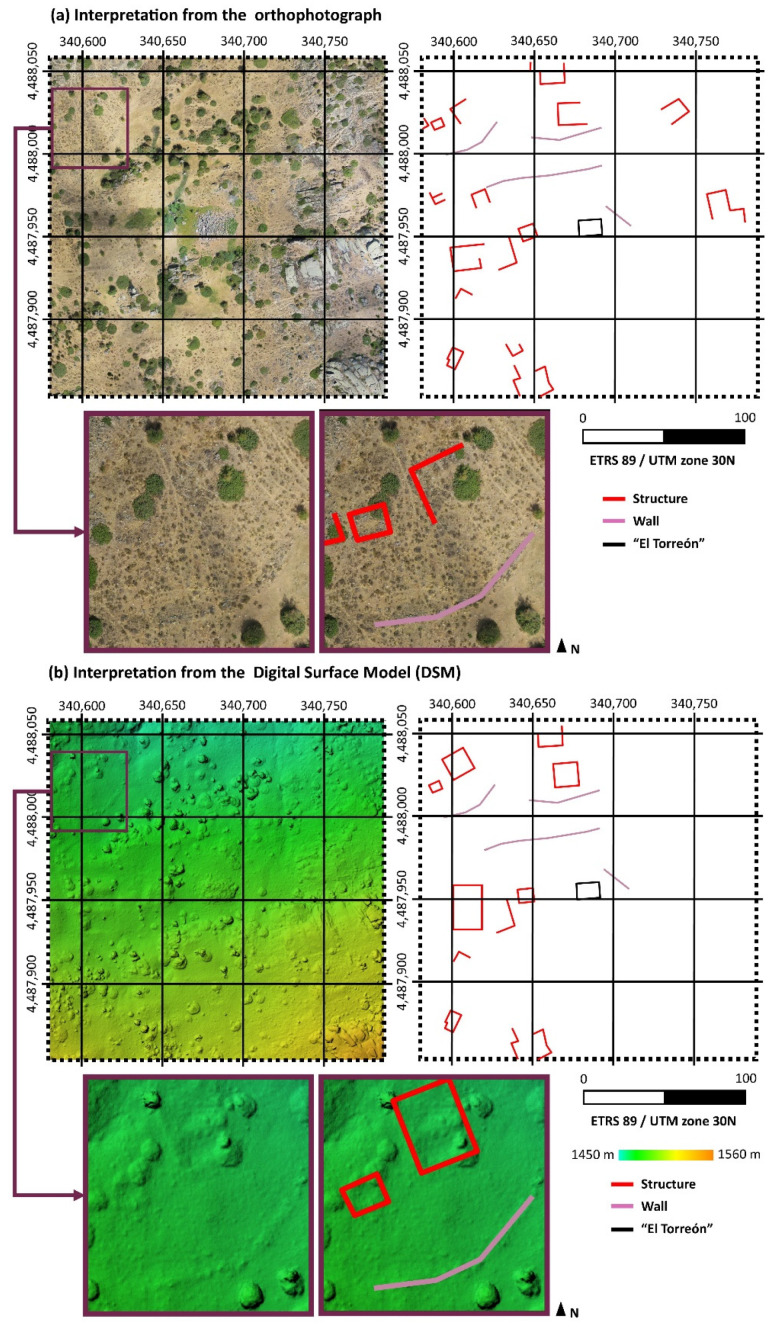
(**a**) Analysis of the orthophoto for the location of elements around “El Torreón”. (**b**) Analysis of the DSM for the location of elements in the area surrounding “El Torreón”.

Once obtained, the products derived from the previous data were then analyzed as an attempt to locate structures around “El Torreón”. Firstly, orthophoto was used. Looking at this product in detail, alignments could be located regarding elements of construction, walls, and other types of unclassified elements in the form of rock alignments. The results obtained are represented graphically in Figure 5a.

Furthermore, the DSM was studied with the aim of finding possible elevations along the z axis that could be useful for the location of structures. These results can be observed in Figure 5b.

As can be seen in the previous Figure 5, results are not identical. Figure 5a is obtained from characteristics such as aligned rocks in the ground, paying special attention to the planimetry. By contrast, Figure 5b shows the differences in height between surfaces, that is to say, it is focused on the altimetry. From the study, a GNSS with centimeter accuracy was used to locate all the structures shown in Figure 5.

#### 3.1.2. Characterization at Feature Scale (Detailed Model)

**Figure 6:**
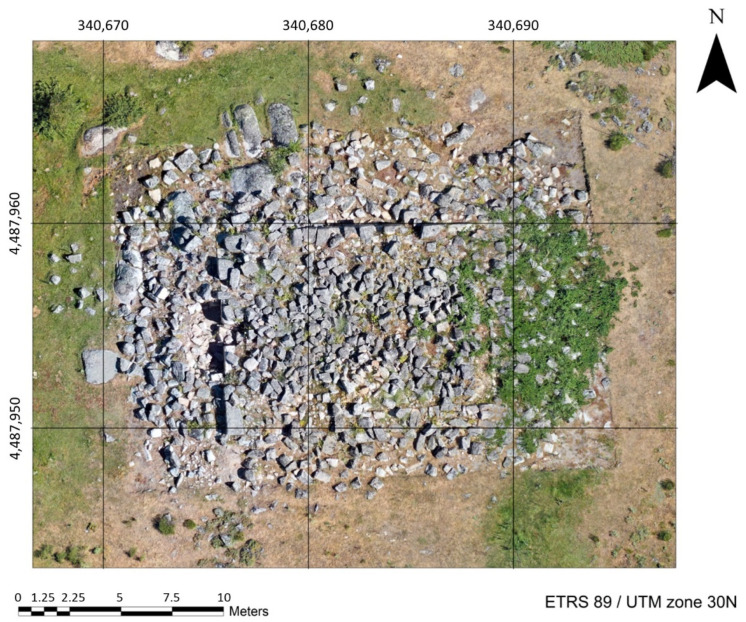
Detailed orthophoto of “El Torreón”.

Once the products derived from data collection were obtained, orthophoto was used to determine the measurements, orientation, and details of these ruins (Figure 6). Through this, it was possible to verify that the construction actually measures 14 m × 9.9 m (length × width), occupying an area of approximately 140 m^2^. Likewise, in some points, exterior collapse of the structure exceeds 5 m. Furthermore, “El Torreón” has a preferential east–west orientation, although slightly turned a few degrees to the northwest (approximately 4 sexagesimal degrees).

### 3.2. Inner Characterization of “El Torreón”

#### 3.2.1. Magnetometry

**Figure 7:**
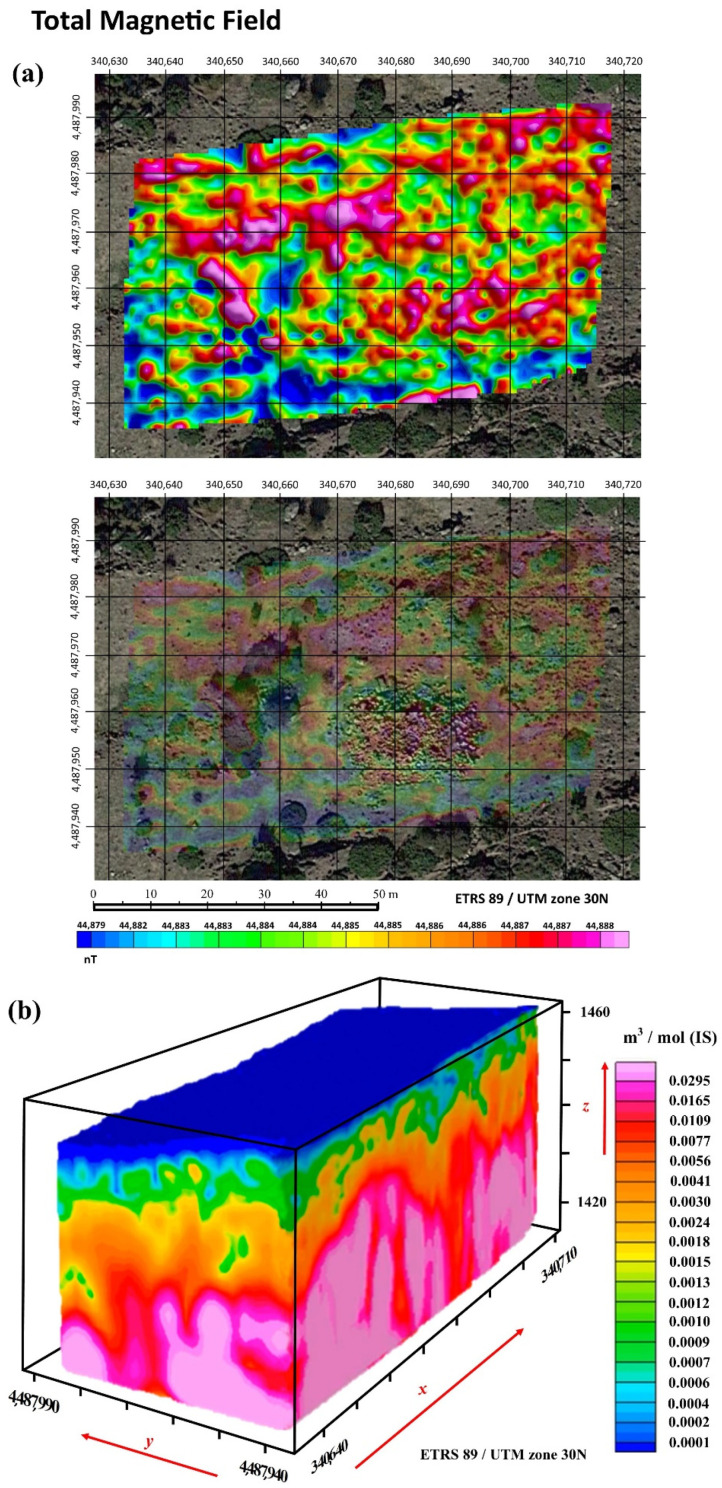
Total magnetic field. (**a**) Surface magnetometric map with representation of the total magnetic field at depth (nT). (**b**) Magnetometric 3D map with representation of the magnetic susceptibility at depth (magnetic susceptibility), logarithmic scale.

Magnetic data were subjected to reductions of the International Geomagnetic Reference Field (IGRF) to obtain the anomaly of the total magnetic field. In addition to this, a series of specific mathematical enhancement processing routines was also applied for obtaining the final results. Figure 7 presents the surface total magnetic field (Figure 7a) and 3D magnetic susceptibility at depth (Figure 7b).

**Figure 8:**
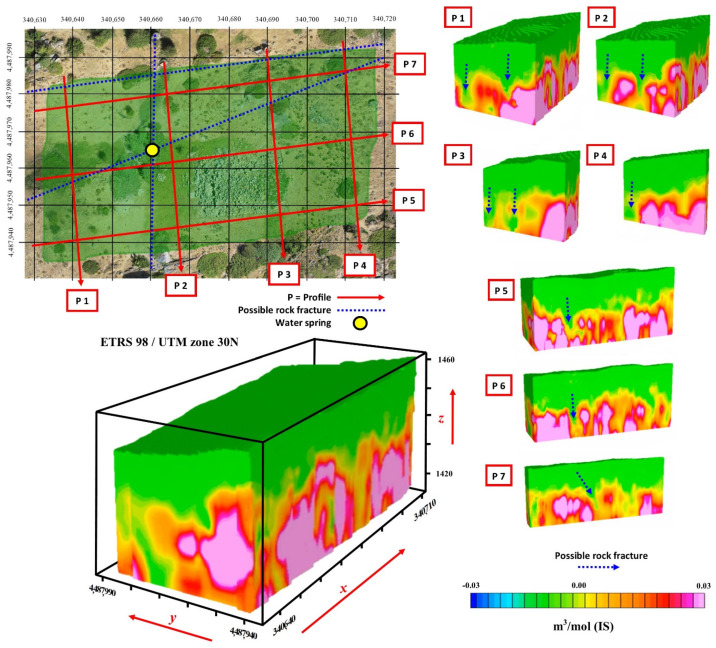
Results derived from the magnetometric 3D (magnetic susceptibility), Gaussian scale.

Figure 8 on the other hand presents the magnetometric 3D. From the corresponding results derived from the magnetometric 3D (magnetic susceptibility), performed with Voxi Earth Modelling algorithm of Oasis Montaj Software and geological data, it can be seen how the local geology corresponds to medium-coarse grain biotitic adamellites (porphyritic facies), a type of granite formation. Furthermore, this particular methodological approach has been able to obtain data from approximately 40 m below surface, both in the 3D block and in the different profiles obtained from this information (Figure 8). From here, the upper areas (in green) can be seen to present slight alterations of the granite through less magnetic susceptibility, eventually leading to the appearance of more compact granites with greater magnetic susceptibility (in red) (Figure 8). From this perspective, analysis of the seven profiles of the 3D block (Figure 8) can reveal certain areas where the altered granite enters the most compact granites. These anomalies reflected in the 3D block correspond to fractures in the more compact granite due to movements or faults that usually serve as a channel through which groundwater circulates. Thus, as the orthophoto shows, there are certain areas where there is humidity represented by vegetation, which, by comparing them with the geophysical study, correspond to the fracture areas. Furthermore, it can be seen that the spring that emanates in the northwest part of “El Torreón” corresponds to the cut zone between two fractures (Figure 8, study interpretation; yellow circle).

#### 3.2.2. Ground Penetrating Radar

**Figure 9:**
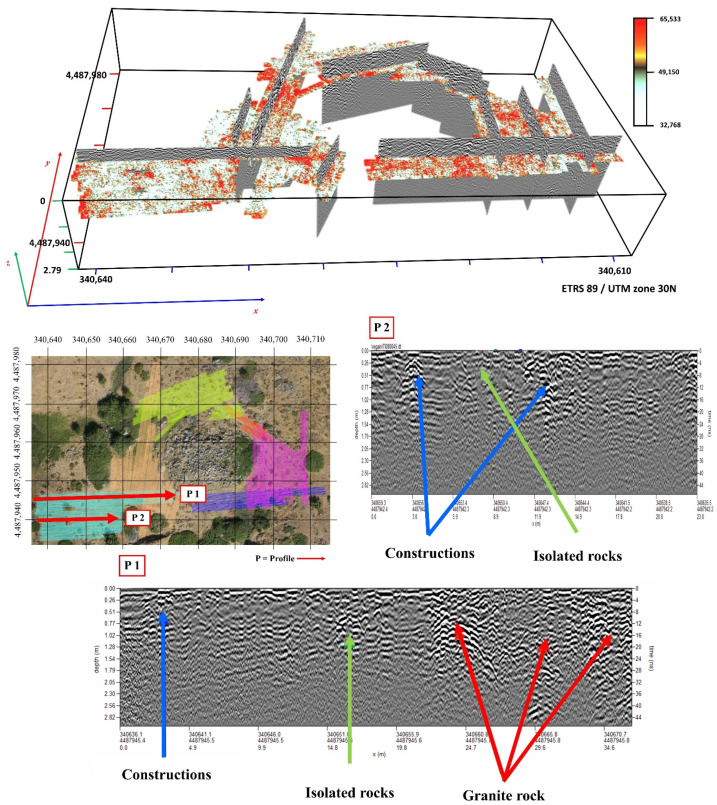
GPR 3D blocks with horizontal slices and vertical sections. Radargrams with interpretations of the anomalies.

With the data obtained from vertical sections, five 3D blocks of data were made by interpolation with the weighted inverse distance method. Data can be viewed on a series of horizontal slices for planned observation of wave amplitudes. The wave amplitudes transformed by the processing gain that have formed the plane of the structures or anomalies of the study area are presented in red (Figure 9).

Through the different radargrams, diverse reflection typologies can be observed (Figure 9). Hyperbola groupings with great signal amplitude are detected. These present alignments both vertically and horizontally and have consequently been interpreted as areas of contact between the granite rock, the soil, and its internal structures, such as joints or fractures with some moisture content.

**Figure 10:**
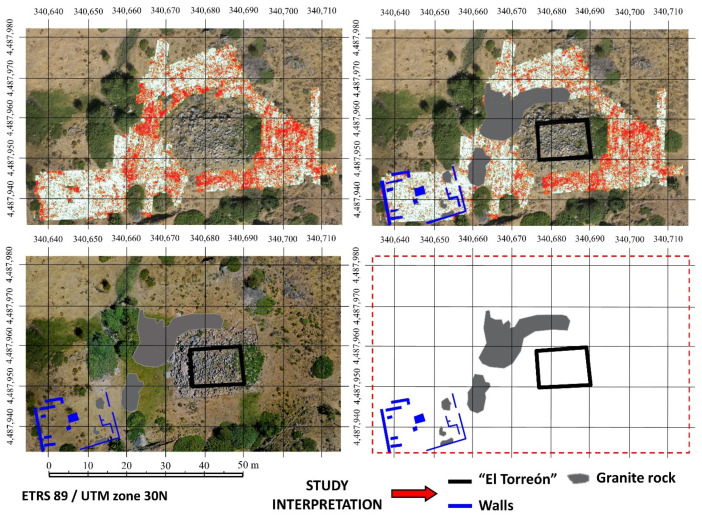
GPR results and interpretative maps.

In the SW area from “El Torreón”, hyperbolic-type anomalies with a lower signal amplitude and wall typologies have been detected. The horizontal sections present certain alignments confirming that they are most probably walls. Occasionally, at these depths (ca. 20 cm), scattered and grouped anomalies have also been detected, which are consequently interpreted as point elements that could be the product of large objects (Figure 10).

## 4. Discussion

**Figure 11:**
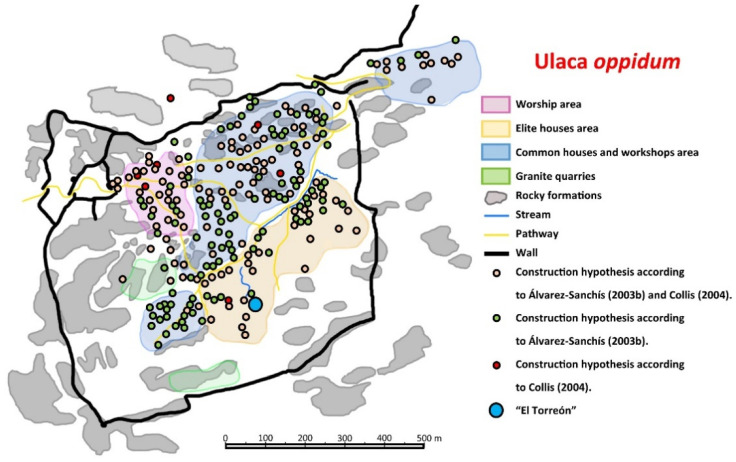
Plan showing the distribution of the different constructions of the Ulaca *oppidum* resulting from the analysis of the main plans obtained from several previous investigations [46,51].

Most of the methodologies used in the present work (see Figure 5 and Figure 10) suggest that “El Torreón” is placed in a more densely inhabited area of the town than previously thought (Figure 11) [46,51]. Thus, it is known now that there are several structures around “El Torreón”, especially to the west, some of them large. This fact may be because “El Torreón” is located in a privileged place inside the *oppidum*, a raised platform from which a large part of the city and the Amblés Valley can be seen. This location and the initial height of the building (taking into account the large number of blocks that are part of its collapse) have led different researchers to support a possible defensive function for these ruins [38] (p. 415). In this sense, it is worth mentioning the similarity of proportions between “El Torreón” and some defensive towers built by the Iberian peoples between the V–IV centuries BCE, namely those of the northeastern Iberian Peninsula: Alorda Park (Calafell, Tarragona, Spain), Puig de Sant Andreu (Ullastret, Gerona, Spain), and Burriac (Cabrera de Mar, Barcelona, Spain). The first two towers measure 7.9 m × 5.6 m (length × width), while the last one measures 5.9 m × 4.19 m. Unfortunately, it is not possible to know how tall these towers were. In any case, the dimensions of these structures are adjusted to the proportion √2, that is, the result of dividing the length by the width of the construction is 1.414 [68]. The same happens in the case of “El Torreón” in Ulaca, which, as has already been mentioned, measures 14 m × 9.9 m. The use of this geometric approach system is most likely due to the fact that it would be the simplest one for architects when proposing rectangular solutions [68].

**Figure 12:**
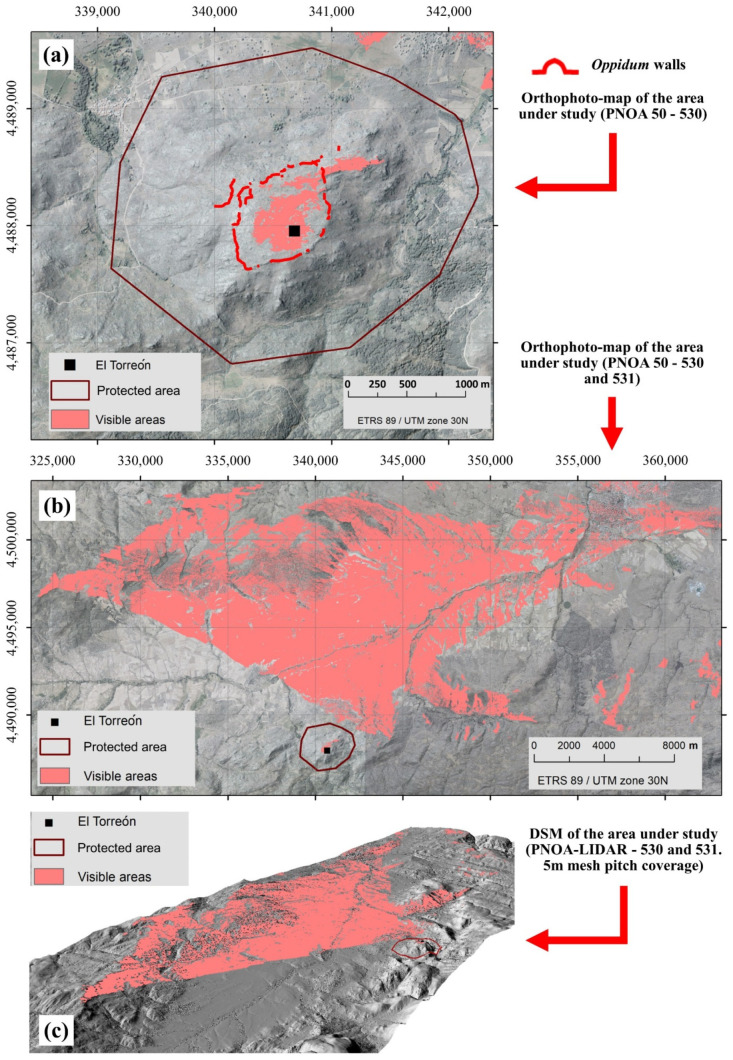
(**a**) Representation of the viewshed analysis of “El Torreón” over the interior of the town. (**b**) Representation of the viewshed of “El Torreón” over the Amblés Valley. (**c**) Three-dimensional representation of the viewshed of “El Torreón” over the Amblés Valley.

With the aim of verifying the effectiveness of “El Torreón” as a possible watchtower, Figure 12 represents the results of a viewshed analysis conducted over the interior of the town (Figure 12a), as well as over the Amblés Valley (Figure 12b,c), based on the assumption that the building had a hypothetical height of about 6 m. This height corresponds to that of the highest structure preserved in the Vettonian area; the walls of the hillfort of “Los Castillos” (Gema, Yecla de Yeltes, Salamanca), which are 6 m high [51] (p. 133). In any case, other viewsheds have been calculated assuming different heights for the Tower (4 m, 8 m, and 10 m) and the results do not change significantly. In the first of these images (Figure 12a), it can be seen that “El Torreón” visually controls a large extension of the inhabited area of the town. It is also noteworthy that only some of the buildings located in the area of worship, namely those buildings in the extreme northwest of the *oppidum*, have no direct sight of this structure due to an elevation located in this sector of the town. Similarly, in the second and third of the aforementioned figures (Figure 12b,c), the enormous viewshed of “El Torreón” over the whole of the Amblés Valley can be verified, especially in the northern edge, constituted by the mountain ranges surrounding Ávila. Despite this, “El Torreón” lacks visual control over certain areas of the extreme southeast and southwest of the valley due again to the elevation situated in the northwest area of the *oppidum*. This would partially limit its usefulness as a watchtower, putting into question this particular interpretation.

For obtaining the previous figures, the locations of the digital surface model that are visible from “El Torreón” have been determined. The visibility of each cell is calculated by comparing two angles from “El Torreón”: the altitude angle towards the center of the cell and the altitude angle towards the local horizon. This last angle is obtained by considering the existing terrain between the observation point (“El Torreón”) and the center of each cell. A point is considered visible when it is above the local horizon. All the above has been calculated by using the viewshed tool of software ArcGIS. Additionally, for the calculation of the viewshed, the height considered was the terrain surface in the MDT, adding 6 m (considering the hypothetical height of around 6 m of “El Torreón”). Other calculations with heights of 4 m, 8 m, and 10 m were also performed, showing that differences were almost imperceptible.

**Figure 13:**
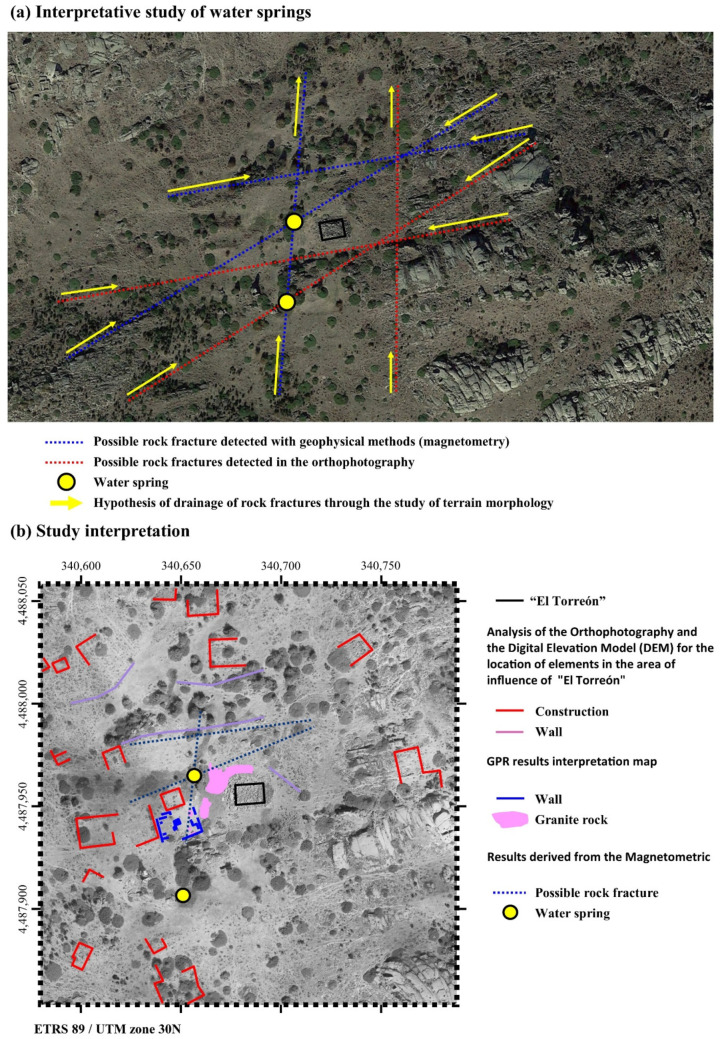
Images showing the hypothesis based on the results of the study. (**a**) Possible rock fracture detected with geophysical methods and orthophoto. (**b**) Location of other large buildings near “El Torreón”.

A second relevant aspect about the location of “El Torreón” is its proximity to one of the springs inside the *oppidum* which never dries out, not even in the harshest moments of summer. Today, in this area there is an apparently modern fountain formed by several large granite blocks, which possibly come from the collapse of the Tower or another nearby structure. This spring, together with another which emerges at a higher elevation close by, takes advantage of the existing fractures in the rock, as well as cracks that have been detected empirically through magnetometry and intuitively through orthophoto when observing patterns in vegetation (Figure 13a). The close link between “El Torreón” and the springs can be explained by the importance of water for the survival of the inhabitants of the town and their livestock, especially during the summer when some of the springs are usually dry. This may have motivated the need to temporarily control its access through construction of an imposing structure. A similar strategy can be observed in the tower of Hijovejo (Quintana de la Serena, Badajoz), a structure chronologically associated with the later years of the Roman Republic where a bastion was built to hide and protect a spring as well as provide general defense to the site [69]. Another possibility suggested by author Álvarez-Sanchís [51] (pp. 150–151) alludes to the importance of springs and fountains in the organization of space within the *oppida*, as is clear in the case of the monumental basin of Bibracte (Mont Beuvray, Burgundy, France), which seems to constitute the town’s urban and ideological center [70]. Additionally, the use of lustral water in the rites of Celtic tradition, alongside the possible existence of a water divinity in Ulaca, could explain the construction of “El Torreón” at this specific vantage point.

Finally, it is necessary to highlight the location of other large constructions near “El Torreón” (Figure 13b). These buildings have been detected thanks to: the identification of stone alignments through orthophoto (Figure 5a), the identification of elevations along the z axis through DSM (Figure 5b), and the detection of permittivity contrasts through GPR (Figure 10). The accumulation of large structures in this area alongside the greater proportions (about 120 m^2^ of useful surface) and the best construction system of “El Torreón” have led different researchers to suggest a public use for this building [41] (p. 28) [47]. According to Fernández-Götz [71,72], the selection of hilltop sites, such as Ulaca, would be a response to religious motivations, considering how this type of naturally uneven topography is not ideal for daily life. This author proposes that the site could have been “an ancestral meeting place in the summer pastures prior to the establishment of the settlement” [72] (p. 143). In this sense, “El Torreón” could have fulfilled a complementary political-religious function to the one carried out by the sanctuary. In this way, it could constitute the monumentalization of an old space of social aggregation, becoming the place where the meetings of the council of elders or nobles of the *oppidum* would take place [53] (p. 234).

## 5. Conclusions

This paper presents a multidisciplinary methodological approach to the study of the ruins and the area surrounding the large building known as “El Torreón”. Thus, the present study uses remote sensing and geophysical techniques to provide a surface and underground view of the characteristics of the building and its surroundings. This multidisciplinary methodological approach has provided greater knowledge of the place or element that will be the subject of future interventions, revealing geometric, morphological, and geological data from a more integrated perspective. This approach has also been successful in providing a higher resolution in the documentation of the site without the need for destructive or invasive interventions through excavation. In this way, the analysis has found a variety of surface structures with drone imagery, at least one building with GPR, and possibly the location of a groundwater channel with magnetometry.

The research performed the digital documentation of “El Torreón” and its surroundings as far as possible. However, at the current state of research, as the structure remains unexcavated, it is not possible to provide a definitive archaeological interpretation of its function. In any case, this study highlights that the different techniques applied have allowed us to refine the interpretive hypothesis. In this sense, the effectiveness of “El Torreón” as a possible watchtower has been verified for the first time by performing a viewshed analysis. In addition, the discovery of new structures around “El Torreón”, in some cases large, contributes to glimpsing the importance of this area of the settlement, where buildings with a possibly political-religious function were erected. In addition, the fractures in the rock detected by the magnetometer, together with the nearby springs, provide valuable data to understand the location of the Tower in this privileged zone from a hydrological point of view.

The results of this work will guide future interventions that will be carried out in the area of “El Torreón”. Under this premise, the 3D models obtained from this study can map out the location of the blocks both inside and outside the structure. This documentation first presents a tool that can be used to plan the movement, removal, and transfer of the blocks, a necessary step for excavation to continue. Likewise, based on data derived from GPR in the immediate area of “El Torreón”, the location of future excavations can be planned to investigate the nature of detected anomalies. It could be considered necessary to perform several test pits in the area to confirm or disprove the existence of other structures. It is important to mention that this is the first work combining remote sensing and geophysical prospecting for defining the surface architecture of the site and its contribution will be essential for the development of future research in the field.

Finally, it is necessary to highlight the enormous benefit of multidisciplinary teams in different phases of excavation, restoration, and the study of materials. This is seen in how multidisciplinarity allows for, but is not limited to; (i) the ability to face different problems that arise on a daily basis in an excavation campaign; (ii) the ability to increase decision-making capabilities; (iii) the application of more precise and less invasive excavation methods; (iv) the ability to improve documentation processes for both excavated and undiscovered materials.

## Data Availability

Data sharing not applicable.
